# In vivo confocal microscopy (IVCM) analysis of corneal sub-basal nerve plexus (SNP) and corneal sensitivity after micropulse transscleral cyclophotocoagulation (MP-TSCPC) in glaucoma patients

**DOI:** 10.1007/s10792-025-03862-2

**Published:** 2025-11-24

**Authors:** Ho Ming Wong, Lok Yee Tsui, Shuet Yan Poon, Ka Wai Kam, Noel Ching-yan Chan, Alvin L. Young

**Affiliations:** 1 Department of Ophthalmology and Visual Sciences, Prince of Wales Hospital, Shatin, New Territories, Hong Kong China; 2 Department of Ophthalmology and Visual Sciences, The Chinese University of Hong Kong, Ma Liu Shui, Hong Kong China; 3 Department of Ophthalmology and Visual Sciences, Prince of Wales Hospital, 30 Ngan Shing Street, Shatin, New Territories, Hong Kong China

**Keywords:** Micropulse transscleral cyclophotocoagulation(MP-TSCPC), In-vivo confocal microscopy (IVCM), Cornea, Corneal sensitivity, Corneal nerve density, Neurotrophic keratopathy

## Abstract

**Purpose:**

To investigate the clinical outcome of micropulse transscleral cyclophotocoagulation (MP-TSCPC) and its effect on corneal sensitivity, as well as corneal nerve parameters using in vivo confocal microscope (IVCM) in eyes with glaucoma.

**Patients and methods:**

This was a prospective longitudinal study recruiting consecutive glaucoma patients who were scheduled to undergo MP-TSCPC for intraocular pressure (IOP) control in a tertiary center. Pre-operative and postoperative clinical parameters were compared including best-corrected visual acuity, IOP, number of topical glaucoma medication, requirement of oral acetazolamide and corneal sensitivity. Intraoperative and postoperative complications were recorded. Pre-operative and postoperative corneal nerve parameters measured using IVCM were analysed in both operated and non-operated eye. All clinical and imaging parameters were compared longitudinally between baseline, postoperative 2 weeks and 1 month.

**Results:**

Twenty consecutive glaucoma patients who received MP-TSCPC between January 2024 and May 2024 were recruited. Postoperatively, there was a significant reduction in IOP (*p* = 0.000) and oral acetazolamide use (*p* = 0.001) while visual acuity were maintained (*p* = 0.710). However, there were reduced corneal sensitivity (*p* = 0.014), decrease in corneal nerve fibre density (CNFD) (*p* = 0.017) and corneal nerve branch density (CNBD) (*p* = 0.047) as detected by IVCM from baseline to 1 month in the operated eye.

**Conclusion:**

MP-TSCPC is an effective glaucoma treatment in terms of IOP control and reduction of glaucoma medications. However, it may potentially result in reduced corneal sensitivity, corneal nerve fiber density and branch density at postoperative 1 month.

## Introduction

Cyclophotocoagulation (CPC) is a laser cycloablative procedure aiming to reduce aqueous secretion and hence intraocular pressure (IOP) through destruction of ciliary body epithelium and stroma and it was introduced for managing eyes with refractory glaucoma since 1970s. [1, 2] Transscleral CPC (TSCPC) is the most common mode of diode laser delivery while the energy delivery pattern can be classified into two categories: continuous wave (CW)-TSCPC and micropulse (MP)- TSCPC. Unlike CW-TSCPC, MP-TSCPC applies short bursts of energy (0.5 ms) followed by rest periods (1.1 ms), allowing selective coagulative necrosis in pigmented tissue while preserving non-pigmented tissue from attaining coagulative threshold[1]. MP-TSCPC has been shown to be an effective IOP lowering treatment with a better safety profile in terms of postoperative morbidity as seen in CW-TSCPC [1]. Thus, apart from being a treatment option for refractory glaucoma alone, MP-TSCPC has been gaining popularity for managing patients with earlier stage of glaucoma especially for those who are unfit to undergo filtration surgery [2, 3].

Among different postoperative complications of TSCPC, neurotropic keratitis (NK) has occasionally been reported after both CW-TSCPC and MP-TSCPC in patients with or without predisposing risk factors for reduced corneal sensation. [4–6]. As laser energy was applied to the ciliary body transsclerally, it was hypothesized that the long posterior ciliary nerve running in the suprachoroidal space near the target area can be damaged. This may lead to cornea denervation [7], and thus a loss of protective mechanism and trophic support resulting in potentially sight threatening NK [8].

In recent years, in vivo confocal microscopy (IVCM) has been a useful tool for assessing, diagnosing and monitoring different corneal diseases [9–12], there were yet limited reports investigating corneal nerve alteration using IVCM after TSCPC not to mention longitudinal study [7]. Thus, we conducted this prospective study aiming to evaluate any change in corneal sensitivity and potential alternation in corneal sub-basal nerve plexus (SNP) morphology using IVCM for glaucoma patients receiving MP-TSCPC.

## Methods

### Study design

This is a prospective longitudinal study evaluating the effect of MP-TPCPC on corneal sensitivity and corneal SNP parameters using IVCM in eyes with glaucoma. The study was in compliance with the tenets of the Declaration of Helsinki and was approved by the joint Chinese University of Hong Kong-New Territories East Cluster Clinical Research Ethics Committee (2023.505). All patients were recruited from the tertiary ophthalmology clinic in the Prince of Wales Hospital in Hong Kong with written informed consent obtained.

### Inclusion and exclusion criteria

Consecutive adult Chinese patients with glaucoma, regardless of type or stage, who were scheduled to undergo isolated MP-TSCPC for IOP control at the Prince of Wales Hospital Ophthalmology Clinic were recruited from January 2024 to May 2024.

Exclusion criteria including: 1) patients receiving combined procedure (i.e. cataract extraction with MP-TSCPC), 2) patient who were unable to provide informed consent, and cooperate with clinical examination and/or in-vivo confocal microscopy, 3) patients who had history of receiving major keratorefractive or corneal surgeries (i.e. corneal transplant), or other ocular surgery except laser peripheral iridotomy, laser trabeculoplasty or cataract extraction, intracranial surgery, central nervous system or systemic abnormalities affecting the function of trigeminal ganglia or peripheral nerves.

### Study measurements and follow up

All patients were recruited and examined by one principal investigator [H.M.W.]. Demographic data and past ocular history were collected at baseline. Clinical and imaging parameters for both study and contralateral eyes were collected at baseline and during follow up visits at 2 weeks and 1 month after MP-TSCPC. Clinical parameters included best-corrected visual acuity (BCVA) as measured by Snellen Chart, slit-lamp biomicroscopy examination, IOP measurement by Goldmann applanation tonometry (GAT), number of topical glaucoma medications and requirement of oral acetazolamide. Details of corneal sensitivity measurement and IVCM were as follows.

### Corneal sensitivity

Corneal sensitivity of the central cornea was measured using Cochet-Bonnet esthesiometer prior to any application of topical anaesthesia. The fully extended thin nylon monofilament was retracted in 5 mm increments until the patient could feel its touch on the central cornea. Result was recorded upon repeated measurement reaching the same value twice.

### In vivo confocal microscopy (IVCM)

IVCM (Heidelerg Retina Tomograph III Rostock Cornea Module) was performed on both eyes after the measurement of corneal sensitivity. For each eye, speculum was inserted after instillation of one drop of topical tetracaine hydrochloride 0.5%. A sterile, single-use disposable cap (TomoCap) was used after application of coupling agent. The subbasal nerve plexus (SNP) was imaged using validated method as described by Takhar et al. [13] A 5 × 5 dot grid system was placed in front of the fellow eye of the patient. Central whorl of the SNP was first identified after asking patient to fixate at the target red dot on the system. Patients were then instructed to look along the grid system from left to right, row by row. One image was captured at each dot and thus at least 25 images were acquired per eye.

ACCMetrics, a validated fully automatic corneal nerve analysis software, was then used to analyse the SNP images [14]. A total of seven parameters were generated including[10]: (1) corneal nerve fibre density (CNFD)- number of fibres per mm^2^, (2) corneal nerve branch density (CNBD)- number of branch points on the main fibres per mm^2^, (3) corneal nerve fibre length (CNFL)- total length of nerves in mm per mm^2^, (4) corneal nerve fibre total branch density (CTBD)- total number of branch points per mm^2^, (5) corneal nerve fibre area (CNFA)- total nerve fibre area mm^2^ per mm^2^, (6) corneal nerve fibre width (CNFW)- average nerve fibre width mm per mm^2^, and (7) corneal nerve fibre fractal dimension (CNFrD)- a measure of corneal nerve complexity. The mean value of all 25 images in each eye was used for analysis.

### MP-TSCPC procedure

Prior to the application of laser, the operative eye received retrobulbar injection of anaesthesia with Atkinson needle and 3 ml of combined 2% lignocaine and 0.5% bupivacaine (1:1). Eye opening was maintained using Katena Double-X Speculum V-wire during the laser application. MPCPC was performed with IRIDEX Cyclo G6 ® (Moutain View, CA) using the MicroPulse ® P3 probe at the power of 2500 mW with an operating cycle of 31.3%. The probe was applied perpendicularly to the globe surface at least 1.5 mm posterior to the limbus as per instructions of the operative manual. Throughout the treatment duration, the surgeon applied the probe to the treatment area using a sweeping action from 10 o’clock to 2 o’clock (superior hemisphere) and 4 o’clock to 8 o’clock (inferior hemisphere) avoiding 3 o’clock and 9 o’clock at a rate of 20 s per hemisphere. The total treatment duration was determined clinically by the surgeon according to the preoperative IOP, target IOP and previous response to MPCPC if any. After the operation, patients were prescribed topical dexamethasone 0.1% and chloramphenicol 0.5% four times daily for 14 days. They were followed up at 1 day, 2 weeks and 1 month postoperatively. Topical glaucoma medications and oral acetazolamide were continued and titrated according to IOP measurements during postoperative visits.

### Statistical analysis

All statistical calculations were performed using IBM SPSS statistics version 24 with statistical significance set at 5% (*p* = 0.05). Snellen BCVA was converted to LogMAR for statistical analysis. Continues variables were reported by mean and standard deviation while categorical data were reported by frequency. Normality was assessed by direct visualization of data histogram. Difference in baseline demographic were compared using Chi-square test, while for corneal sensitivity and IVCM parameters, paired T-test were used to compare between study eye and non-operated eye at baseline, and between individual time points with its baseline. Repeated measures analysis of variance (ANOVA) was used to examine across different time points.

## Results

### Demographics

**Fig. 1:**
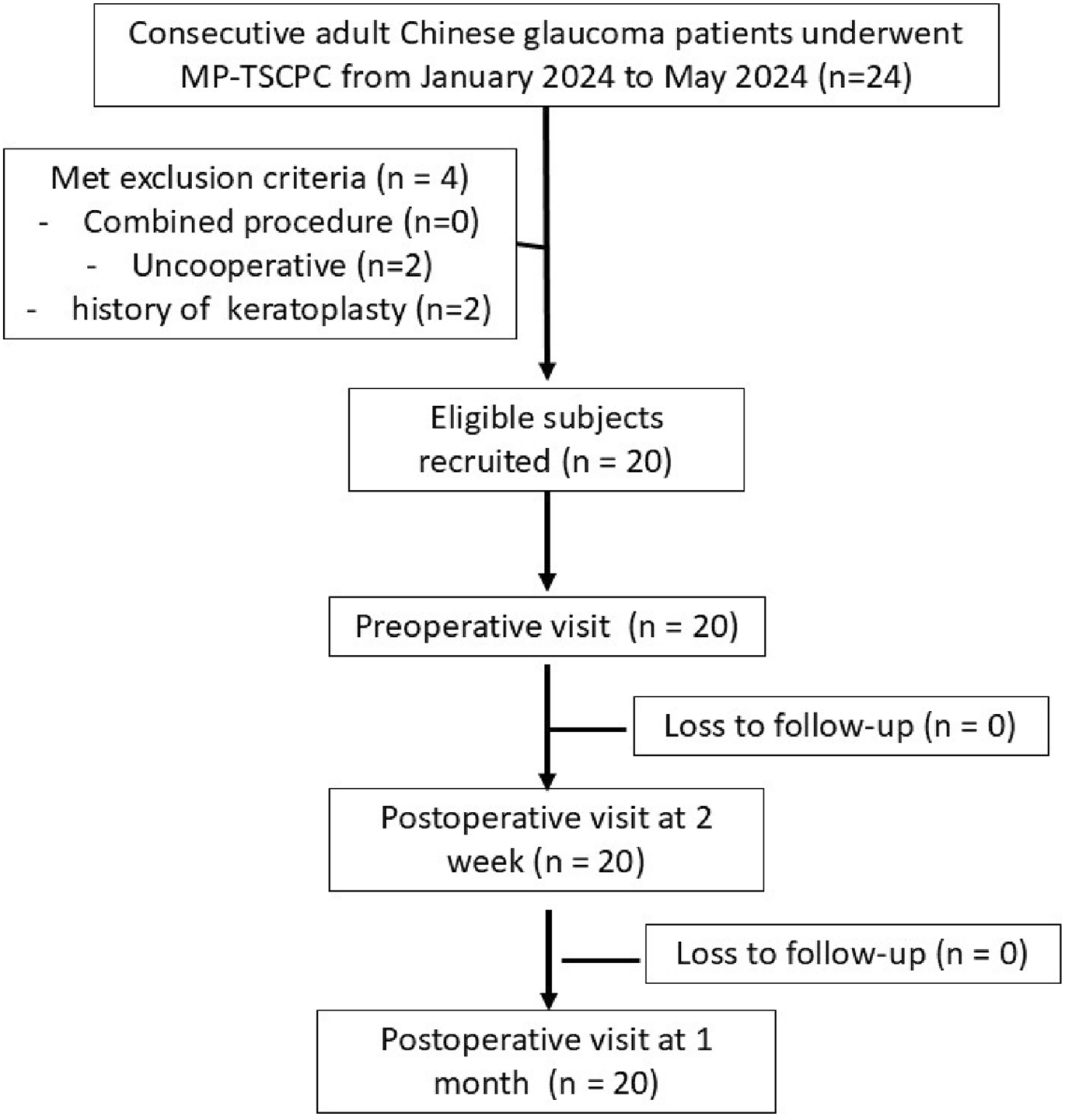
Study flowchart of patient selection and inclusion. MP-TSCPC = Micropulse-Transscleral Cyclophotocoagulation

Twenty-four patients underwent MP-TSCPC during the study period and 20 patients (14 males (70%), 6 with unilateral glaucoma (40%)) were recruited (see Fig. 1) with a mean age of 65.75 ± 8.90. MP-TSCPC was performed on the right eye in 10 (50%) patients and all recruited subjects completed three visits as per study requirement. The demographics and etiologies of glaucoma (if any) for the operated and non-operated eye were summarized in Table 1.

**Table 1:** Demographics of operated and non-operated eye in recruited patients with GON

Age (years old)	65.75 ± 8.902	
Gender (male)	70%	
Laterality of operated eye (right)	50%	
	Operated eye	Non-operated eye
Etiology of glaucoma		
ICE syndrome	1	0
NTG	1	1
NVG	3	0
PACG	3	3
POAG	6	6
SO induced glaucoma	2	1
Traumatic glaucoma	2	0
Uveitic glaucoma	2	1
No glaucoma	0	8

ICE syndrome: Iridocorneal Endothelial syndrome; NTG: Normal Tension Glaucoma; NVG: Neovascularization Glaucoma, PACG: Primary Angle Closure Glaucoma; POAG: Primary Open Angle Glaucoma; SO induced: Silicone Oil induced

### Baseline clinical characteristics and IVCM parameters

Baseline clinical characteristics and IVCM parameters were summarized in Table 2. Operated eyes had a statistically significant lower baseline BCVA (*p* = 0.000), higher baseline IOP (*p* = 0.000) and topical glaucoma medication load (*p* = 0.001) when compared to non-operated eye. However, there was no statistically significant difference in corneal sensitivity (*p* = 0.110) at baseline. Among all IVCM parameters, operated eyes had lower baseline values of CNFD, CNBD, CNFL and CTBD (*p* = 0.032, *p* = 0.0027, *p* = 0.018, *p* = 0.045), while other IVCM parameters did not show a statistically significant difference.

**Table 2:** Baseline clinical characteristic and IVCM parameters between operated and non-operated eye

	Operated eye	Non-operated eye	*P* value
BCVA (LogMAR)	1.66 ± 0.852	0.424 ± 0.703	0.000*
IOP (mmHg)	27.35 ± 10.951	14.75 ± 3.385	0.000*
Number of topical anti-glaucomatous medications	4.05 ± 0.510	2.30 ± 2.105	0.001*
Number of previous CPC			0.368
0	9	19	
1	5	0	
2	4	1	
3	0	0	
4	1	0	
Corneal sensitivity (mm)	55.75 ± 5.911	57.50 ± 4.443	0.110
IVCM parameters			
CNFD (/mm^2^)	9.38 ± 5.703	13.99 ± 7.168	0.032*
CNBD(/mm^2^)	13.28 ± 11.63	23.84 ± 17.44	0.027*
CNFL(mm/mm^2^)	8.43 ± 3.65	11.42 ± 3.99	0.018*
CTBD(/mm^2^)	29.87 ± 21.00	45.87 ± 29.08	0.045*
CNFA(mm^2^/mm^2^)	0.0052 ± 0.0022	0.0063 ± 0.0021	0.077
CNFW(mm/mm^2^)	0.02 ± 0.01	0.2285 ± 0.0015	0.186
CNFrD	1.408 ± 0.120	1.46 ± 0.362	0.061

BCVA: best-corrected visual acuity; CNFD: corneal nerve fibre density; CNBD: corneal nerve branch density; CNFL: corneal nerve fibre length; CPC: cyclophotocoagulation; CTBD: corneal nerve fibre total branch density; CNFA: corneal nerve fibre area; CNFW: corneal nerve fibre width; CNFrD: corneal nerve fibre fractal dimension; IOP: intraocular pressure; IVCM: in vivo confocal microscopy

^*^p value ≤ 0.05

### Clinical efficiency of MP-TSCPC

The mean total energy delivered to the operated eye by MP-TSCPC was 203.84 ± 41.05 J. After MP-TSCPC, there was a significant reduction in IOP (27.35 ± 10.95 mmHg to 16.10 ± 4.47 mmHg, *p* = 0.000) as well as mean oral acetazolamide requirement (1.42 ± 1.29 tab to 0.55 ± 0.83 tab, *p* = 0.001) at postoperative 1 month. The BCVA and the number of topical glaucoma medications remained unchanged at 1 month (*p* = 0.710, *p* = 0.330). Details were summarized in Table 3. There was no intraoperative and postoperative complication including NK reported during the follow up period.

**Table 3:** BCVA, IOP, number of topical anti-glaucomatous medication, oral acetazolamide and corneal sensitivity at 2 weeks and 1-month post-TS-MPCPC in operated eye

	Baseline	2 weeks	1 month	*P* value
BCVA (logMAR)	1.66 ± 0.85	1.66 ± 0.84	1.63 ± 0.86	0.710
IOP (mmHg)	27.35 ± 10.95	15.40 ± 5.33	16.10 ± 4.47	0.000*
Number of topical anti-glaucoma medications	4.05 ± 0.510	4.05 ± 0.510	3.95 ± 0.510	0.330
Oral acetazolamide	1.42 ± 1.29	1.025 ± 1.36	0.55 ± 0.83	0.001*
Corneal sensitivity (mm)	55.75 ± 5.91	53.00 ± 7.50	52.50 ± 5.96	0.049*

BCVA: best-corrected visual acuity; IOP: intraocular pressure

^*^p value ≤ 0.05

### Progressive reduction in corneal sensitivity in operated eye after MP-TSCPC at 2 weeks and 1 month

Overall significant reduction of corneal sensitivity in the operated eyes were demonstrated at 1 month after MP-TSCPC (*p* = 0.049) as shown in Table 4. There was a significant reduction in corneal sensitivity in operated eye between baseline and postoperative 2 weeks (*p* = 0.003), baseline and postoperative 1 month (*p* = 0.014), postoperative 2 weeks and postoperative 1 month (*p* = 0.016). However, there was no changes in corneal sensitivity along different time points of follow up visits for the non-operated eyes as shown in Table 4.

**Table 4:** Corneal sensitivity and IVCM parameters in operated eye and non-operated eye at postoperative 2 weeks and 1 month

		Operated eye			Non-operated eye		
			*P* value (vs. baseline)	*P* value (vs. 2 weeks)		*P* value (vs. baseline)	*P* value (vs. 2 weeks)
Corneal sensitivity (mm)	Baseline	55.75 ± 5.91			57.50 ± 4.44		
	2 weeks	53.00 ± 7.50	0.003*		56.50 ± 4.32	0.385	
	1 month	52.50 ± 5.96	0.014*	0.016*	56.25 ± 3.93	0.330	0.772
CNFD (/mm^2^)	Baseline	9.38 ± 5.70			13.99 ± 7.17		
	2 weeks	6.45 ± 4.13	0.075		15.22 ± 8.00	0.852	
	1 month	5.58 ± 5.24	0.017*	0.536	14.25 ± 6.20	0.779	0.671
CNBD(/mm^2^)	Baseline	13.28 ± 11.63			23.84 ± 17.44		
	2 weeks	9.94 ± 9.10	0.252		25.84 ± 20.26	0.725	
	1 month	7.72 ± 7.78	0.047*	0.267	20.70 ± 11.13	0.170	0.376
CNFL(mm/mm^2^)	Baseline	8.43 ± 3.65			11.42 ± 3.99		
	2 weeks	7.85 ± 3.99	0.519		12.09 ± 4.80	0.870	
	1 month	6.65 ± 4.07	0.065	0.266	10.99 ± 3.33	0.190	0.335
CTBD(/mm^2^)	Baseline	29.87 ± 21.00			45.87 ± 29.08		
	2 weeks	29.74 ± 23.46	0.810		49.52 ± 32.32	0.767	
	1 month	23.09 ± 15.49	0.173	0.196	38.20 ± 17.62	0.048*	0.187
CNFA(mm^2^/mm^2^)	Baseline	0.005 ± 0.002			0.006 ± 0.002		
	2 weeks	0.01 ± 0.004	0.793		0.01 ± 0.003	0.741	
	1 month	0.004 ± 0.003	0.359	0.263	0.001 ± 0.002	0.160	0.243
CNFW(mm/mm^2^)	Baseline	0.02 ± 0.01			0.23 ± 0.001		
	2 weeks	0.02 ± 0.006	0.449		0.02 ± 0.001	0.510	
	1 month	0.02 ± 0.009	0.703	0.319	0.002 ± 0.002	0.606	0.819
CNFrD	Baseline	1.4 ± 0.12			1.46 ± 0.36		
	2 weeks	1.34 ± 0.36	0.485		1.46 ± 0.07	0.433	
	1 month	1.22 ± 0.47	0.063	0.229	1.45 ± 0.40	0.064	0.806

CNFD: corneal nerve fibre density; CNBD: corneal nerve branch density; CNFL: corneal nerve fibre length; CTBD: corneal nerve fibre total branch density; CNFA: corneal nerve fibre area; CNFW: corneal nerve fibre width; CNFrD: corneal nerve fibre fractal dimension

^*^p value ≤ 0.05

### Reduction in CNFD and CNBD at 1 months in operated eye after TS-MPCPC

**Fig. 2:**
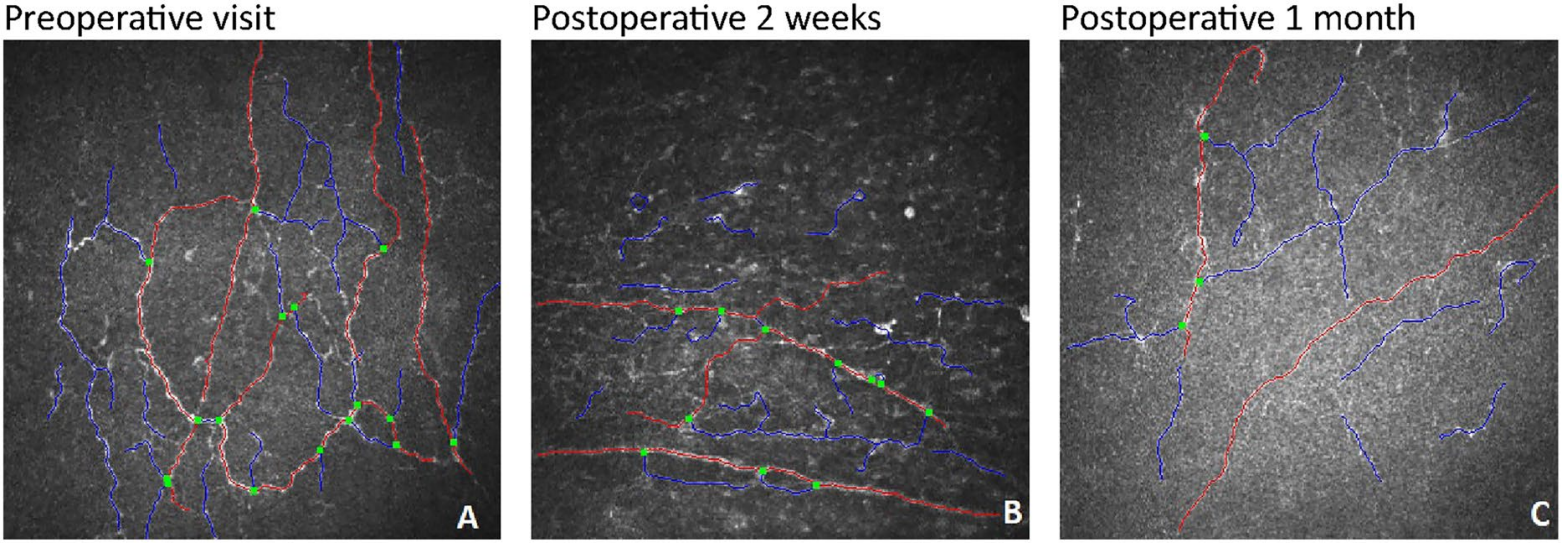
Comparative IVCM images of the same patient’s operated eye preoperatively (**A**), at postoperative 2 weeks (**B**) and at postoperative 1 month (**C**). IVCM = In Vivo Confocal Microscopy Red line = The main corneal nerve fiber. Blue line = A corneal nerve branch Green dot = A point where a nerve branch originates from the main nerve

IVCM parameters across each time points and statistical comparisons were summarized in Table 4. For the operated eyes, as compared to baseline, there was a significant reduction in CNFD (9.38 ± 5.70/mm^2^ to 5.58 ± 5.24/mm^2^, *p* = 0.017) and CNBD (13.28 ± 11.63/mm^2^ to 7.72 ± 7.78/mm^2^, *p* = 0.047) at 1 month after MP-TSCPC. (see Fig. 2) There was no significant change observed in other IVCM parameters and at 2 weeks after MP-TSCPC.

For the non-operated eye, there was no statistically significant change observed between baseline and the 2 postoperative time points except for CTBD which had shown a slight reduction (45.87 ± 29.08/ mm^2^ to 38.20 ± 17.62/ mm^2^, *p* = 0.048) at postoperative 1 month.

## Discussion

Traditional treatment algorithm of glaucoma follows a stepwise approach from topical medications, laser, to surgical interventions, with increasing efficacy and yet interventional risk. Minimally invasive glaucoma surgeries (MIGS) have been gaining popularity in the recent decade and yet their high cost-proficiency ratios have limited their applications in patients with refractory glaucoma. Therefore, TSCPC has remained one of the major glaucoma treatment options especially since the introduction of MP-TSCPC. With the advantages of a better safety profile (less destruction to tissue and less postoperative inflammation), less intraoperative pain, higher repeatability and feasibility as an office procedure, MP-TSCPC had been utilized to treat various types and severity of glaucoma including those of early disease stage. In our centre, we had delivered more than 260 TSCPC procedures in 2021 and a rising trend in demand of this treatment can be observed. Nevertheless, there had been case reports and clinical experience of corneal complications after MP-TSCPC including corneal edema [15] and NK [6, 7].

NK is a sight threatening condition with the hallmark of reduction in corneal sensitivity, spontaneous epithelial breakdown, and impaired epithelial defect healing [16]. This complication had been reported after CW-TSCPC in glaucoma patients with underlying risk factors [5, 17]. There were multiple ocular or systemic conditions associated with decreased corneal sensitivity, including history of herpetic keratitis, chemical burns, corneal dystrophies, prior corneal incision for ocular surgeries, diabetes, multiple sclerosis and leprosy [13]. In patient with glaucoma, chronic use of topical medical therapy can disrupt ocular surface structures leading to reduction in corneal sensitivity as well as alternation in SNP features as demonstrated by IVCM [14, 15, 17, 18]. In our cohort, as compared to the non-operated contralateral eye, the operated eyes were using more glaucoma medication at baseline given their higher IOP. Prior to MP-TSCPC, these eyes also showed significant difference in baseline IVCM parameters (CNBD, CNFL and CTBD) despite similar corneal sensitivity. During subgroup analysis for eyes with unilateral glaucoma, similar reduction of SNP parameters can also be demonstrated at baseline between operated and non-operated eye. This highlighted the potential effect of glaucoma medications being one of the potential culprits in altering corneal innervation and the potential risk of NK development in patients who had been put on multiple glaucoma medications for a prolonged duration.

In contrast to our study, there was no significant change in corneal sensation nor SNP density as measured by IVCM 1 month after CW-TSCPC in the prospective study by Ravio et al. [7]. This discrepancy may be explained by the relatively smaller sample size, lower total energy used and the lack of automatic corneal image assessment in that case series. Another point of note between our study and Ravio et al. also included the difference in energy delivery pattern as CW-TSCPC was applied in a more focal fashion with interval space between laser spots during its application as compared to the sweeping motion during application of MP-TSCPC.

In the present study, there was no clinically manifested neurotropic keratitis identified in any of the operated eyes during the follow-up period. However, even with our relatively small sample size, we noticed significant reduction in corneal sensitivity, corneal nerve fiber density and branching density 1 month after MP-TSCPC. Whether these clinical and imaging findings correlate with increased risk of NK was out of the scope of this prospective study. However, our pilot findings had demonstrated the potential corneal alterations after the application of MP-TSCPC and caution might be taken during case selection and upon application of this procedure in patients with susceptible risk of developing NK.

The present study carried some limitations. First, we included a heterogenous group of glaucoma patients with various etiologies and stages. Also, more than half of the operated eyes have previous history of cyclophotocoagulation procedures ranging between 1 to 5 years ago. This heterogeneity may lead to a different degree in corneal nerve susceptibility and thus responses to this index MP-TSCPC. Second, we have employed the contralateral non-operated eye as control rather than age-matched healthy controls. Nevertheless, corneal sensitivity and SNP parameters were compared at different time points of the same eye for either group. Thirdly, we did not exclude patient with diabetes of which concomitant corneal hypoesthesia may lead to potential confounder during analysis. Lastly, the small sample size in our study limited the use of multi-variable regression analysis, hence we were not able to adjust for potential confounding factors like age and sex in corneal nerve parameters.

As part of the peripheral nervous system, trigeminal nerve also carries regenerative potential and such potential had been demonstrated in patients upon recovery from herpes zoster ophthalmicus [9, 19]and in post-LASIK eyes [12, 20] with recovery duration ranging from 2 months up to 5 years after the initial insult. As our study only demonstrated reduction in corneal sensitivity and SNP parameters at 1 month after MP-TSCPC, it is uncertain whether such difference would persist after longer follow up duration. Further study with a larger sample and longer follow-up duration will be required to determine the long-term effect of corneal alteration after one treatment of MP-TSCPC and if there would be any potential neuronal regeneration after the insult by MP-TSCPC. These data will be important as it may shed light onto the optimal timing of repeated procedure for IOP control in patient with refractory glaucoma.

## Conclusion

MP-TSCPC is an effective treatment in terms of IOP control and medication reduction for various types of glaucoma. However, there was significant reduction in corneal sensitivity, corneal nerve fiber density and branching density 1 month after MP-TSCPC as compared to preoperative baseline.

## Data Availability

The data of this study are available from the corresponding author upon reasonable request.
